# Point-of-Care Ultrasound in Non-obstetric Hemorrhage: A Critical Diagnostic Tool

**DOI:** 10.7759/cureus.94860

**Published:** 2025-10-18

**Authors:** Cheila Batista, Rúben Calaia, Joana Brandão, Elena Segura-Grau

**Affiliations:** 1 Anesthesiology, Unidade Local de Saúde Viseu Dão-Lafões, Viseu, PRT

**Keywords:** hemoperitoneum, hemorrhagic shock, pocus, postpartum hemorrhage, ultrasonography

## Abstract

Postpartum hemorrhage remains a significant contributor to maternal mortality worldwide. While obstetric causes are more common, non-obstetric intra-abdominal hemorrhage is a rare yet potentially life-threatening condition. We present a clinical case of a 35-year-old pregnant woman who underwent an elective cesarean section that subsequently developed hypovolemic shock at the post-anesthetic care unit, due to non-obstetric intra-abdominal hemorrhage. Point-of-care ultrasound proved to be an essential diagnostic tool for identifying free intra-abdominal fluid, emphasizing its importance in early hemorrhage recognition. A multidisciplinary approach involving anesthesiology, obstetrics, and surgery was crucial in resuscitating and stabilizing the patient. Early recognition and intervention are essential in managing non-obstetric postpartum hemorrhage and minimizing associated morbidity and mortality.

## Introduction

Postpartum hemorrhage is the leading cause of maternal morbidity and mortality worldwide, occurring more frequently after cesarean delivery than after vaginal delivery [1]. According to the American College of Obstetricians and Gynecologists, postpartum hemorrhage can be classified as minor or major when blood loss exceeds 500 or 1000 mL, respectively [2]. However, non-obstetric intra-abdominal hemorrhage, although rare, can also occur. Its diagnosis can be challenging and delayed, contributing to significant morbidity and mortality. Cesarean delivery and associated surgical difficulties, along with blood losses exceeding 500 mL during the procedure, are recognized intermediate risk factors for postpartum intra-abdominal hemorrhage [1].

As the diagnosis of postpartum hemorrhage is typically made within two hours after delivery, proper monitoring in the Post-Anesthesia Care Unit (PACU) is crucial. Clinical signs of hypovolemia, such as hypotension, tachycardia, and altered consciousness, along with abdominal pain, should be regularly assessed. However, cardiovascular physiological alterations observed in this particular population may hinder the prompt identification of postpartum hemorrhage. While the presence of vaginal bleeding allows for the identification of the most likely cause of hypovolemic shock, uterine atony, intra-abdominal hemorrhage is difficult to recognize early, resulting in delayed diagnosis. Although obstetric causes are more common, iatrogenic injury to abdominal organs can occur and lead to life-threatening conditions. Computed tomography is considered the gold standard for diagnosing intra-abdominal hemorrhage. However, its drawbacks, including elevated radiation exposure, delayed access, and logistical issues, render it a less optimal choice for urgent cases [3]. Ultrasound has played an increasingly important role as it is cost-effective, radiation-free, and readily available. Although most patients will have some amount of intraperitoneal free fluid after abdominal or pelvic surgery, a significant accumulation may strongly suggest intra-abdominal hemorrhage. Therefore, point-of-care ultrasound (POCUS) allows for the rapid detection of intra-abdominal fluid and the assessment of signs of hypovolemia, such as inferior vena cava diameter and left ventricular motility and dimension. The Trendelenburg position can also aid in detecting free fluid in the hepatorenal and splenorenal spaces [4].

Hence, in cases of high suspicion, POCUS becomes a valuable tool for identifying the cause of hemorrhage [1,3].

We describe a case of a pregnant woman who developed a hemorrhagic shock following an elective cesarean delivery. 

## Case presentation

A 35-year-old woman, 37 weeks pregnant (G4P2), ARh+ blood type, with a prior history of two cesarean deliveries, and no comorbidities, underwent an elective cesarean delivery with bilateral tubal ligation. Preoperative results showed no abnormality (hemoglobin 12.9 g/dL). The procedure, via Pfannenstiel incision, was performed under spinal anesthesia with 8 mg of 0.5% hyperbaric bupivacaine plus morphine 100 µg, lasted for approximately one hour, and was uneventful. To prevent uterine atony, the patient was administered 10 IU (international units) of oxytocin intravenously (IV). At the end of the procedure, the patient was transferred to the PACU. Upon admission, she was eupneic, normotensive (blood pressure (BP) 110/70 mmHg), normocardic (~60 beats per minute (bpm)), with 100 mL of clear urinary output, a well-contracted uterus, and no signs of active bleeding. She initially complained of epigastric discomfort despite maintaining anesthetic sensitive blockade up to T4 level (both pinprick and cold) and complete motor block of the lower limbs. One hour after admission, the patient experienced a hypotensive episode (BP 60/30 mmHg) without tachycardia (~60 bpm). Arterial blood gas analysis, POCUS, and obstetric evaluation were unremarkable. After IV phenylephrine 100 µg administration, hypotension was reversed. 

**Figure 1:**
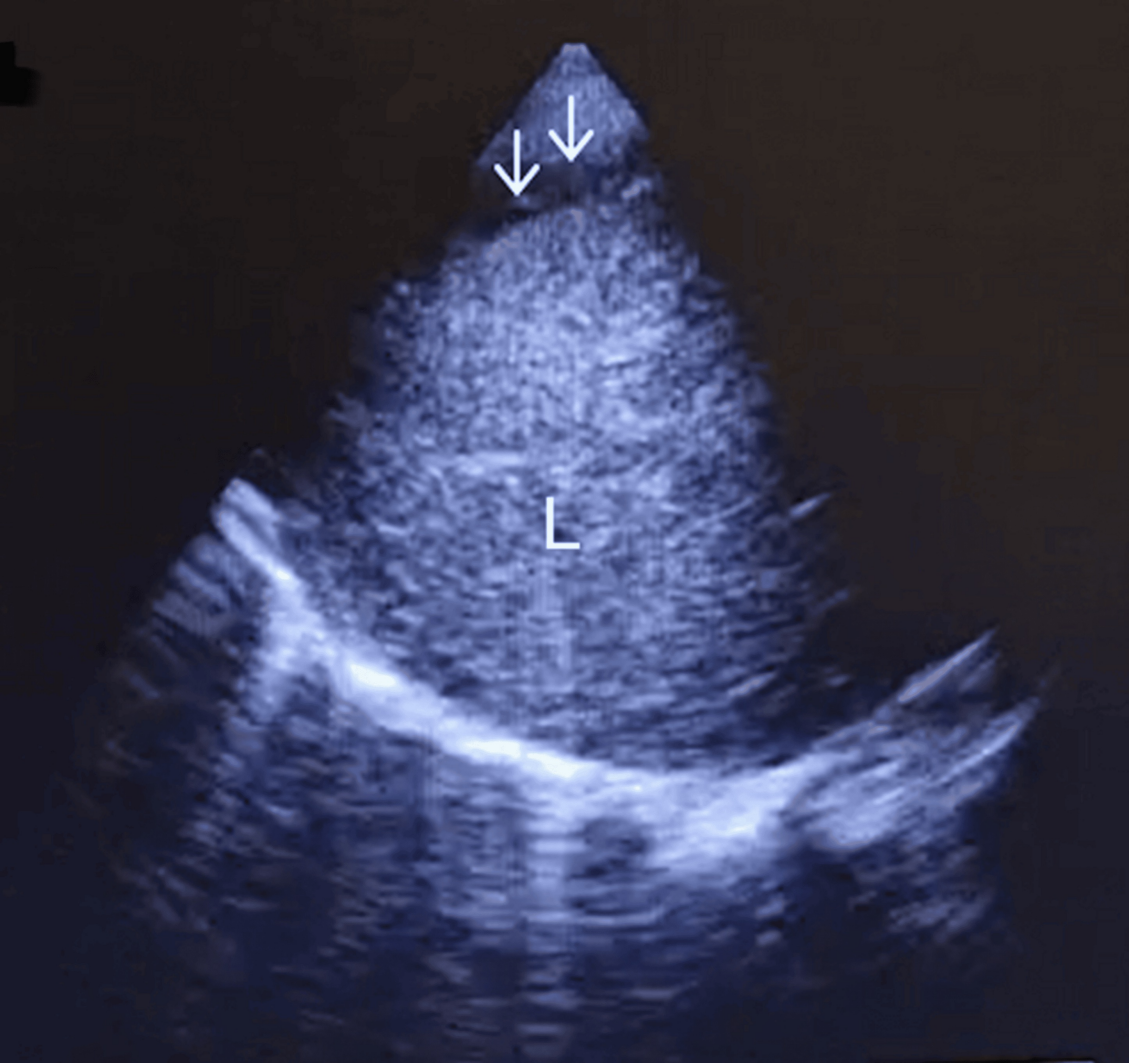
Right quadrant (L, liver; arrows - free intraperitoneal fluid).

**Figure 2:**
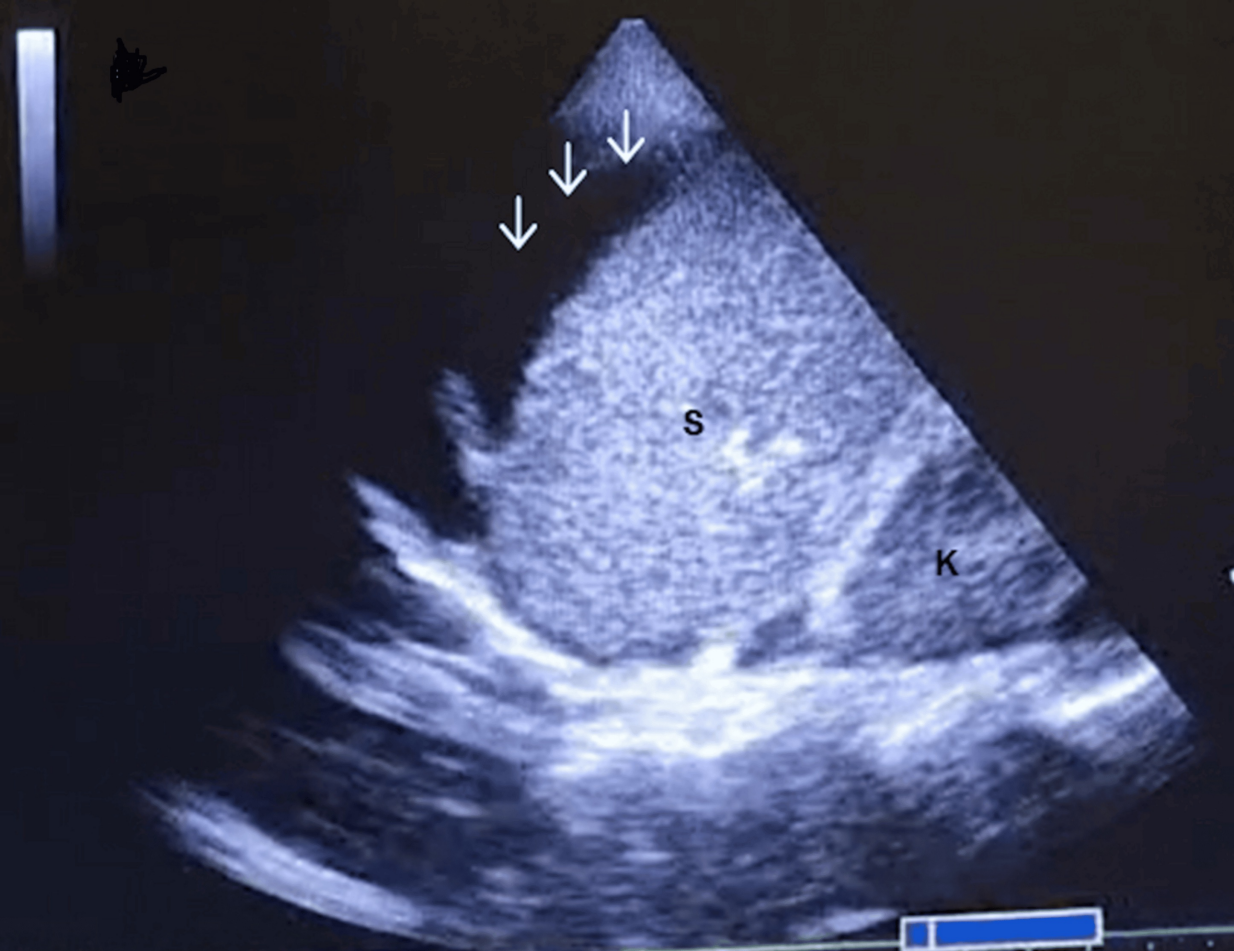
Left quadrant (K, kidney; S, spleen; arrows, free intraperitoneal fluid).

Approximately one hour later, a sudden hypotensive episode (~60/30 mmHg) occurred with sinus tachycardia (~100 to 120 bpm). During abdominal palpation, she exhibited generalized pain and apparent peritoneal irritation. Point-of-care ultrasound (POCUS) was repeated, revealing a significant amount of intraperitoneal free fluid in the right (Figure 1) and left quadrants (Figure 2), as well as suggestive signs of hypovolemia, including a small right ventricle and an inferior vena cava measuring less than 1.5 cm in diameter with a collapsibility index less than 50%.

Arterial blood gas in room air was also repeated, showing the results presented in Table 1.

**Table 1:** Arterial blood gas analysis.

Arterial blood gas parameters (room air)	Values
pH	7.37
Hb	5.9 g/dL
HCO3-	20.9 mmol/L
Ca2+	1.11 mmol/L
Lactate	1.6 mmol/L

**Figure 3:**
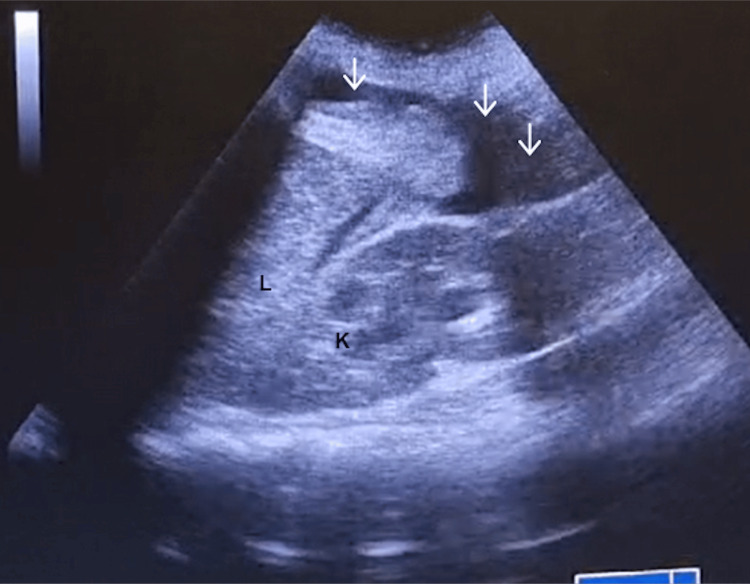
Right quadrant (L, liver; K, kidney; arrows, free intraperitoneal fluid).

Fluid therapy was initiated, along with vasopressors (IV phenylephrine and ephedrine) and Trendelenburg positioning, with a favorable response. The obstetric team was called, and the massive transfusion protocol was activated. Ten minutes later, a follow-up POCUS examination revealed a significantly greater amount of intraperitoneal fluid (Figure 3) compared with the initial scans (Figures 1, 2), indicating worsening hemorrhage and the emergent need for exploratory laparotomy.

This POCUS examination also allowed the anesthetic team to prepare all necessary emergency drugs. Thus, the patient was taken directly to the operating room, reducing wait times. In the operating room, the obstetric team confirmed normal uterine tone, and an emergency exploratory laparotomy was performed.

Upon arrival in the operating room (OR), the patient was monitored according to the American Society of Anesthesiologists (ASA) standards. She presented with a blood pressure of 55/30 mmHg and a heart rate of 135 bpm. Anesthetic induction was performed with IV ketamine 80 mg, IV fentanyl 0.1 mg, and IV suxamethonium 100 mg. During induction, a pulseless electrical activity (PEA) cardiac arrest occurred, requiring IV epinephrine administration (1 mg) to restore sinus rhythm. Massive transfusion protocol was initiated, including the administration of four units of packed red blood cells, two units of fresh frozen plasma, and one platelet pool. Additional treatments included IV fibrinogen 2 g, IV tranexamic acid 1 g bolus, and 1 g in a 6 mL/hour infusion. Anesthesia was maintained with sevoflurane, and IV rocuronium 50 mg was given for neuromuscular blockade maintenance. Phenylephrine infusion was started (50 µg/mL at 60-100 mL/hour) until central venous access was obtained and changed to a noradrenaline infusion at a rate of 0.1 µg/kg/minute. Calcium chloride 1g IV was administered, and a potassium chloride (80 mEq/L) infusion was started. An arterial catheter was placed for invasive BP monitoring, and an esophageal temperature probe was used for core temperature monitoring. Urine output was monitored (1-2 mL/kg/hour). Surgery revealed a rupture of the triangular ligaments of the liver; the patient was left with an open abdomen (laparostomy) and transferred to the intensive care unit (ICU). After 24 hours, she underwent an uneventful surgery for abdominal wall closure. The patient remained in the ICU for eight days, the first three requiring vasopressor support with noradrenaline at a maximum rate of 1.67 μg/kg/min. After noradrenaline suspension, the patient was extubated. Neurological examination at awakening revealed focal neurologic deficits, namely, right hemineglect, right hemiplegia, and right homonymous hemianopsia, and a diagnosis of possible Posterior Reversible Encephalopathy Syndrome (PRES) was made. Initial echocardiographic evaluation at postpartum day 6 showed no contractility abnormalities, but a repeated examination at day 12 revealed a decrease in left ventricular systolic function (EF 65%-45%) with a dyssynchronous pattern of ventricular contraction, and a diagnosis of possible postpartum cardiomyopathy was made. The patient was then started on bisoprolol, candesartan, and dapagliflozin. After nine days of hospitalization in the ICU, the patient remained one week in the ward undergoing physical rehabilitation and obstetric care. The patient was discharged without neurologic deficits and will continue to follow up with cardiology and neurology clinics. 

## Discussion

Post-anesthesia care is crucial for the early detection of complications related to surgical and anesthetic procedures. Non-obstetric postpartum hemorrhage is rare and is a challenging diagnosis that requires a high level of suspicion and is associated with significant morbidity and mortality [5]. These patients may present with abdominal pain, abdominal distension, nausea, vomiting, and hypovolemic shock with anemia. Potential mechanisms for liver ligament rupture include surgical adhesions, prior abdominal trauma, or ligamentous fragility, although no clear predisposing factor was identified in this patient.

Clinical signs of hypovolemic shock may initially be masked by neuraxial anesthesia, resulting in delayed recognition of intra-abdominal hemorrhage in the immediate postoperative period. PACU plays a crucial role in patient resuscitation and stabilization through the administration of fluids, vasopressors, and blood products. Minute zero (MZ) POCUS [6], which refers to the use of ultrasound immediately after delivery in a specific clinical situation, plays a significant role in identifying and managing these complications. An early POCUS scan is crucial in the obstetric population to anticipate causes of shock, like hypovolemic or cardiac shock. Moreover, continuous POCUS evaluation is essential to early detection of complications, allowing for timely interventions that can significantly improve patient outcomes. Additionally, it enhances clinical decision-making by providing immediate feedback on clinical situations or on the effectiveness of treatments, thus facilitating adjustments. While PACU screening protocols could facilitate early recognition of atypical postpartum hemorrhage, their effectiveness is constrained by operator dependency and the need for sufficient training and experience.

The swift action and coordination of emergency interventions are vital to the positive outcome of postpartum hemorrhage. Thus, anesthesiology plays a critical role in patient care in this context, and multidisciplinary decision-making is essential.

## Conclusions

Post-anesthesia care is crucial for the early detection and management of complications after surgery, especially in obstetric patients. Although rare, non-obstetric hemorrhage after cesarean delivery carries a high degree of morbidity and mortality, requiring prompt multidisciplinary intervention.

This case reinforces the value of performing POCUS at the initial clinical evaluation. This rapid, bedside ultrasound technique enables timely diagnosis of life-threatening conditions and facilitates reassessment. In obstetric contexts, such as non-obstetric postpartum hemorrhage, MZ POCUS is a key tool to anticipate or address serious complications.
